# Screnning of viral hepatitis in mental disorder patients: Psiqui-Clinic Programme

**DOI:** 10.1192/j.eurpsy.2022.1221

**Published:** 2022-09-01

**Authors:** M. Cavero, T. Planas, J. Goikolea, S. Lens, C. Bartrés, L. Colomer, C. García, M. Valentí, V. Ruiz, Y. Rivas, A. Benabarre, R. Catalan, G. Masana, J. Colom, X. Forns, R. Martin-Santos, Z. Mariño

**Affiliations:** 1 Hospital Clínic, , department Of Psychiatry And Psychology, Barcelona, Spain; 2 Hospital Clínic, , Liver Unit, Barcelona, Spain; 3 Agéncia de Salut Pública de Catalunya, Programa Pcavihv, Barcelona, Spain

**Keywords:** viral hepatitis, HCV, Severe Mental Disorders, HBC

## Abstract

**Introduction:**

The WHO would increase diagnosis and treatment of viral hepatitis in the world by 2030, based on the high efficacy of direct-acting-antivirals against HCV, extended vaccination programs in HBC, and epidemiological data. Diagnostic of HCV/HBV infection has been simplified by point-of-care (POC) devices (cheap/easy-to-use/interprete/qick-results), detecting anti-HCV-antibodies or HBV-antigen in capillary blood at the patients´site. The current seroprevalence of viral hepatitis B/C in general population in Spain is 0.5%/1% and would be higher (3-17%) in people with severe-mental-disorder due to risk factors and traditionally less access to health care.

**Objectives:**

To design a screening protocol for HCV eradication and HBV-detection, and risk factors among severe-mental-disorder patients in a CommunityMentalHealthCenter. To guarantee equal access to viral hepatitis screening and therapy among this population.

**Methods:**

Outpatients visited along one-year who accepts participate. Using POC-device for qualitative detection of anti-HCV-antibodies (Quickview-of-Lumiquick-Diagnostics®)/HBsAG (Abbott-Rapid-Diagnostics®). Socio-demographic data; mental disorder(ICD-10); HCV/HBV risk-factors; Neurotoxicity-scale (mood/cognition/sleep/gastrointestinal/sickness/motor); SF-12; Patient-satisfaction. Subjects with positive HCV/HBV POC-test will have a on-site venopuncture to assess hemograme/liver tests, and HCV-RNA (Cobas-TaqMan-RocheDiagnostics)/HBsAg-ELISA (Atellica-Siemens). In positive HCV-RNA (active infection) the psychiatric-team will inform the hepatology-team for non-invasive liver fibrosis assessment and DAA prescription. The patient will receive 8-12-weeks on-site treatment, and assessed (Neurotoxicity/SF-12).HCV cure will be confirmed by HCV-RNA in blood. Chronic-cases will be managed at Hepatology-Unit.

**Results:**

We will present the results of the implementation of the programme and their ability to detect viral-hepatitis-positive cases among patients with severe-mental-disorders and to treat them effectively.

**Conclusions:**

Our results may support the generalisation of the programme in among CMHC’s.

**Disclosure:**

No significant relationships.

